# Supplementary material for: Structural Dynamics, Phonon Spectra and Thermal Transport in the Silicon Clathrates
Source: Molecules. 2022 Sep 29;27(19):6431. doi: 10.3390/molecules27196431 (PMC9572326; doi:10.3390/molecules27196431)
Supplement: Supplementary file 1 [file molecules-27-06431-s001.zip › molecules-1916441-supplementary.pdf]

Supplementary Materials: Structural Dynamics, Phonon Spectra and Thermal Transport in the Silicon Clathrates

Benxiang Wei, Joseph M. Flitcroft and Jonathan M. Skelton

1. Additional Calculations on the C-II Framework Structure

As discussed in the text, we found that our calculations on the Clathrate-II (C-II) structure did not match a number of previous computational studies.[1–4] In particular, our calculations predict the C-II structure to be $\sim$4 $\times$ higher in energy than bulk diamond Si (d-Si) than the studies in Refs. [1] and [2], and they predict the scalar averaged lattice thermal conductivity κ_{latt} to be around an order of magnitude smaller than the calculations in Refs. [3] and [4].

To investigate these discrepancies further, we performed additional calculations on the C-II structure and, where necessary, on d-Si as a reference point. Since the other studies used the LDA, PW91 or PBE functionals,[5–7] rather than the PBEsol functional used in the present work,[8] we performed calculations with the LDA and PBE functionals.[5,7] (We previously found PW91 and PBE to give very similar results.[9]) Since it is not clear whether the calculations in some of the previous studies were performed on the primitive or conventional unit cells of the two structures, we performed calculations on both. Our initial model for the C-II structure was obtained from the Materials Project (MP) database (**mp-16220**).[10] Although this cites as its source the structure published with Ref. [1] in the Inorganic Crystal Structure Database (**ICSD: 56721**), to check for potential issues during the import and MP workflow we also performed a set of PBEsol calculations starting from the ICSD structure. Finally, to investigate the possibility of technical errors, we performed an additional set of PBEsol calculations on the primitive cell with enhanced convergence criteria, *viz.* a 1.5 $\times$ higher plane-wave cutoff and $\boldsymbol{k}$-point meshes with around 8 $\times$ the number of sampling points. We note that different projector augmented-wave (PAW) pseudopotentials[11,12] were used for the LDA and PBE/PBEsol calculations, so the former also serve as a test for possible issues with the Si pseudopotentials.

Since the studies in Refs. [1] and [2] quote bulk moduli, instead of performing "direct" optimisations of both the atomic positions and cell shape/volume we calculated the energy as a function of volume by performing a series of constant-volume geometry optimisations and fit the resulting $E(V)$ curves to the Birch-Murnaghan equation of state (EoS):[13,14]

$$E(V) = E_0 + \frac{9V_0B_0}{16}\left\{\left[\left(\frac{V_0}{V}\right)^{\frac{2}{3}} - 1\right]^3 B_0' + \left[\left(\frac{V_0}{V}\right)^{\frac{2}{3}} - 1\right]^2 \left[6 - 4\left(\frac{V_0}{V}\right)^{\frac{2}{3}}\right]\right\} \tag{1}$$

where V_0 and E_0 are the equilibrium volume and total energy, respectively, and B_0 and B_0' are the bulk modulus and its pressure derivative.

The calculated $E(V)$ curves and fits are presented in Figures S1-S5. From the fit parameters we obtain from each set of calculations the equilibrium volume V, lattice constant a, bulk modulus B and pressure derivative B' of the d-Si and C-II structures, and the energy difference ΔU_{latt}, all of which are collected in Table S1.

The calculated volume of V = 20.08 Å^3 atom^{-1} and lattice constant of a = 4.536 Å obtained from the PBEsol EoS fits for d-Si are identical to the values obtained by ("direct") geometry optimisations (c.f. Table 2 in the text). The calculated V = 23.03 Å^3 atom^{-1} and a = 14.631 obtained for the C-II structure differ slightly from the V = 23.02 Å^3 atom^{-1} and a = 14.629 Å obtained by optimisation but the differences are negligible. The energy differences ΔU_{latt} of 32.61 and 32.71 kJ mol^{-1} atom^{-1} calculated using the primitive and conventional cells, respectively, are likewise very close to the 32.62 kJ mol^{-1} atom^{-1} calculated between the optimised primitive cells.

The calculated unit-cell volumes and lattice constants are consistent with previous computational studies. Ref. [1] quotes LDA unit-cell volumes of 19.65 and 22.65 Å^3 atom^{-1} for d-Si and C-II, respectively, which are in line with the values in Table S1. Similarly, our LDA lattice constants are similar to the values of 5.42 and 14.57 Å quoted in Ref. [4]. Our predicted PBE volumes for d-Si and C-II are again similar to the PW91/PBE values of 20.38/20.45 and 23.49/23.50 Å^3 given in Refs. [1]/[2], while our lattice constants are compatible with the values of 5.47 and 14.74 Å quoted in Ref. [3]. On the other hand, whereas our calculated bulk moduli for d-Si are compatible with the values of 94.5 (LDA),[1] 90.94 (PW91)[1] and 87.7 GPa (PBE),[3] our values for the C-II structure are 15-20 % smaller than the values of 81.15 GPa (LDA),[1] 75.80 (PW91)[1] and 76.9 GPa (PBE).[2] On the other hand, the Materials Project database entry for the C-II structure (**mp-16220**) lists a PBE volume of 23.54 Å^3 atom^{-1} and a bulk modulus of 62 GPa, which are very close to the PBE values in Table S1. Furthermore, taking the calculated energy per atom for the C-II structure and bulk diamond Si (**mp-149**) gives a ΔU_{latt} of 32.57 kJ mol^{-1} atom^{-1}, which is very close to our PBE value of 32.8 kJ mol^{-1} atom^{-1}.

Taken together, these comparisons suggest that the discrepancy most likely arises from a difference in the atomic positions in the C-II structures used in previous studies and that present in the MP database and used in this work. However, as noted above the MP structure is derived from the ICSD structure published with Ref. [1], and we found that PBEsol calculations on the ICSD structure give near-identical results to those performed on the MP structure (c.f. Table S1). We note that **ICSD: 56721** is the only Si Clathrate II structure recorded in the ICSD, and we were unable to find data in Refs. [2–4] that would allow us straightforwardly to reconstruct the C-II structures used in these studies.

Finally, the $E(V)$ curves for the two primitive cells using tighter convergence criteria give identical unit-cell volumes and lattice constants, very similar B/B', and a very similar energy difference to the PBEsol calculations using the "standard" convergence criteria, which confirms that our chosen technical parameters are adequately converged.

As noted in the text, the thermal-conductivity calculation on C-II reported in Ref. [4] used the (single) conventional cell to determine the second-order force constants (FCs) $\mathbf{\Phi}^{(2)}$, which is smaller than the $2 \times 2 \times 2$ expansion of the primitive cell used in the present work, and also imposed a 4.2 Å cutoff on the third-order FCs $\mathbf{\Phi}^{(3)}$. To investigate whether this could account for the discrepancy with our calculations, we recalculated the $\mathbf{\Phi}^{(2)}$ in the conventional cell and performed a second calculation of the phonon dispersion and density of states (DoS) curve and the lattice thermal conductivity. We also performed a third and fourth calculation of the κ_{latt} in which we used the larger supercell to obtain the $\mathbf{\Phi}^{(2)}$ but imposed range cutoffs of 4.2 and 5 Å on the $\mathbf{\Phi}^{(3)}$.

A comparison of the dispersion and DoS curves obtained with the two sets of $\mathbf{\Phi}^{(2)}$ (Figure S6) shows very little difference, suggesting that both give similar harmonic phonon spectra. The thermal conductivity computed with the $\mathbf{\Phi}^{(2)}$ computed in the conventional cell is ∼10 % (0.66 W m^{-1} K^{-1}) larger at T = 300 K than that computed using the $\mathbf{\Phi}^{(2)}$ determined using the larger supercell. It is therefore unlikely that the smaller supercell used to compute the harmonic force constants could account for the much larger κ_{latt} obtained in the calculations in Ref. [4]. Interestingly, whereas Ref. [4] indicates that truncating the third-order interaction at the second nearest-neighbour distance of 4.2 Å is not expected to impact the κ_{latt}, in our calculation we find that using the same cutoff reduces the calculated thermal conductivity by ∼50 %. Using a larger cutoff of 5 Å to account for the possibility of a longer predicted bond length in this study increases the κ_{latt}, but the value is still ∼30 % smaller than that obtained without truncating the interaction range. On the other hand, the study in Ref. [3] obtained a similar κ_{latt} to that in Ref. [4] but used a larger third-order supercell and included third-order force constants up to sixth nearest neighbours. This provides further evidence that the discrepancies between the present work and previous calculations are very likely due to differences in the structures used.

In summary, these additional calculations confirm that the higher relative energy of the C-II structure predicted by our calculations is robust to the choice of functional and

Table S1. Summary of the equation of state (EoS) calculations performed on the bulk diamond Si (d-Si) and Clathrate II (C-II) structures. For each calculation the table lists the parameters obtained from the Birch-Murnaghan EoS fits (Equation 1; Figures S1-S5), *viz.* the volume per atom V, the lattice constant a, the bulk modulus B and pressure derivative B', and the root-mean-square (RMS) error of the fit, together with a calculation of the lattice energy difference ΔU_{latt}.

		V (Å^3 atom^{-1})	a [Å]	B [GPa]	B'	RMS (10^{-3} meV atom^{-1})	ΔU_{latt} (kJ mol^{-1} atom^{-1})
LDA, prim. cell	d-Si	19.72	5.403	96.71	4.327	2.64	-
	C-II	22.62	14.544	67.45	4.287	1.62	33.87
LDA, conv. cell	d-Si	19.72	5.403	96.62	4.298	1.12	-
	C-II	22.62	14.544	67.45	4.274	2.07	33.87
PBE, prim. cell	d-Si	20.44	5.468	88.89	4.300	2.56	-
	C-II	23.55	14.740	61.58	4.182	1,76	32.80
PBE, conv. cell	d-Si	20.44	4.568	88.90	4.269	1.06	-
	C-II	23.55	14.740	61.61	4.167	2.16	32.80
PBEsol, prim. cell	d-Si	20.08	5.436	93.70	4.285	2.23	-
	C-II	23.03	14.631	65.56	4.230	1.87	32.61
PBEsol, conv. cell	d-Si	20.08	5.436	93.66	4.257	9.08	-
	C-II	23.03	14.631	65.57	4.216	2.12	32.71
PBEsol, prim. cell,	d-Si	20.08	5.436	93.70	4.285	2.23	-
**ICSD: 56721**[1] [1]	C-II	23.03	14.631	65.56	4.249	1.16	32.61
PBEsol, conv. cell,	d-Si	20.08	5.436	93.66	4.257	9.08	-
**ICSD: 56721**[1] [1]	C-II	23.03	14.631	65.58	5.225	1.31	32.71
PBEsol, prim. cell,	d-Si	20.08	4.536	93.63	4.280	2.14	-
tight convergence	C-II	23.03	14.631	65.51	4.223	1.75	32.71

[1] The d-Si values for these comparisons are repeated from the PBEsol calculations on the primitive and conventional cells listed above.

technical parameters employed in the calculations, and suggest that the discrepancy with previous modelling studies is most likely due to differences in the structures used. If this is the case, then the implication is that the structure published with Ref. [1] (**ICSD: 56721**) may not reflect the structures used in this and other modelling studies. We have also confirmed that our thermal conductivity calculations appear to be robust to the choice of supercell used to calculate the second-order force constants, and so we also attribute the differences in the calculated κ_{latt} to the possible differences in the structure. Despite the discrepancy, as noted in the text our calculations confirm that the structure is dynamically stable, if energetically metastable, and thus represents a potential phase of Si, and comparison of the calculated and measured κ_{latt} suggests it perhaps better reflects the structure obtained in the experiments in Refs. [15] and [16].

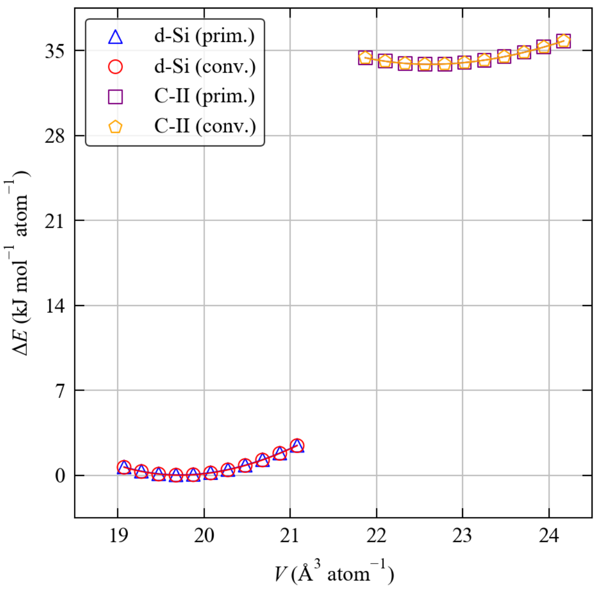

Figure S1. Energy/volume curves for the bulk diamond Si (d-SI) and Clathrate-II (C-II) structures calculated using the LDA functional. Calculations were performed on both the primitive (prim.) and conventional (conv.) cells of each structure. The markers show the calculated data points and the solid lines are fits to the Birch-Murnaghan equation of state (Equation 1).

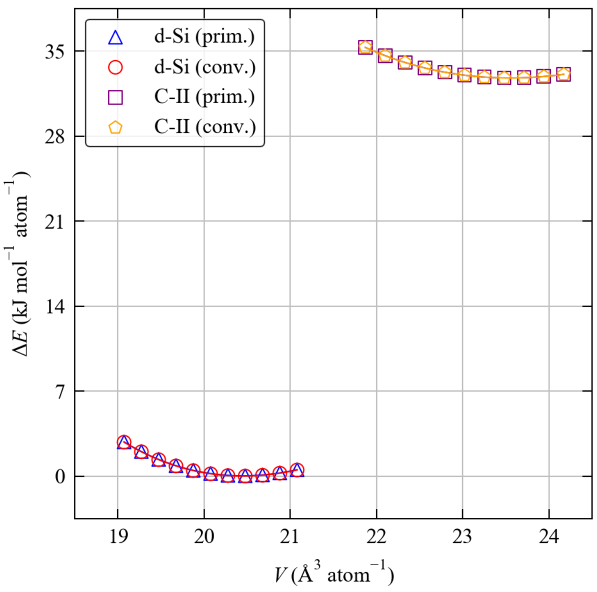

Figure S2. Energy/volume curves for the bulk diamond Si (d-SI) and Clathrate-II (C-II) structures calculated using the PBE functional. Calculations were performed on both the primitive (prim.) and conventional (conv.) cells of each structure. The markers show the calculated data points and the solid lines are fits to the Birch-Murnaghan equation of state (Equation 1).

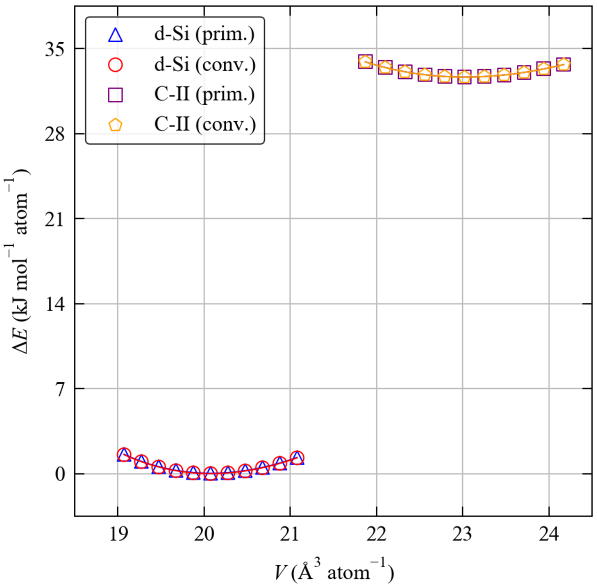

Figure S3. Energy/volume curves for the bulk diamond Si (d-SI) and Clathrate-II (C-II) structures calculated using the PBEsol functional. Calculations were performed on both the primitive (prim.) and conventional (conv.) cells of each structure. The markers show the calculated data points and the solid lines are fits to the Birch-Murnaghan equation of state (Equation 1).

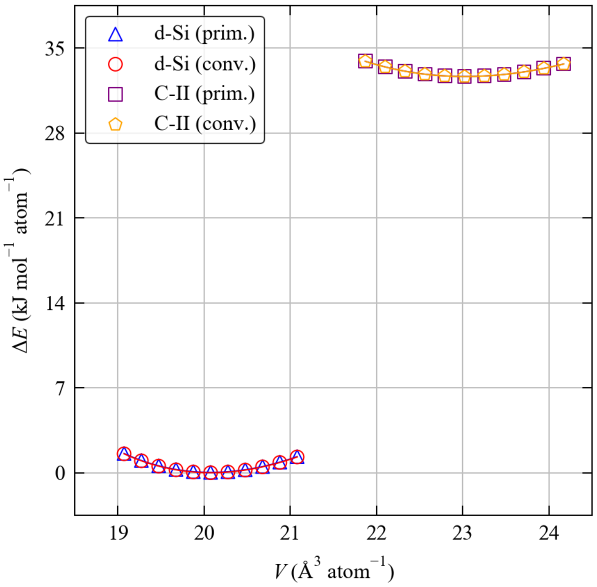

Figure S4. Energy/volume curves for the bulk diamond Si (d-SI) and Clathrate-II (C-II) structures calculated using the PBEsol functional. The C-II structure is that taken from the Inorganic Crystal Structure Database (**ICSD: 56721**),[1] which is listed as the source of the C-II structure in the Materials Project database (**mp-16220**). Calculations were performed on both the primitive (prim.) and conventional (conv.) cells of each structure. The data for the d-Si structure is the same as that shown in Figure S3. The markers show the calculated data points and the solid lines are fits to the Birch-Murnaghan equation of state (Equation 1).

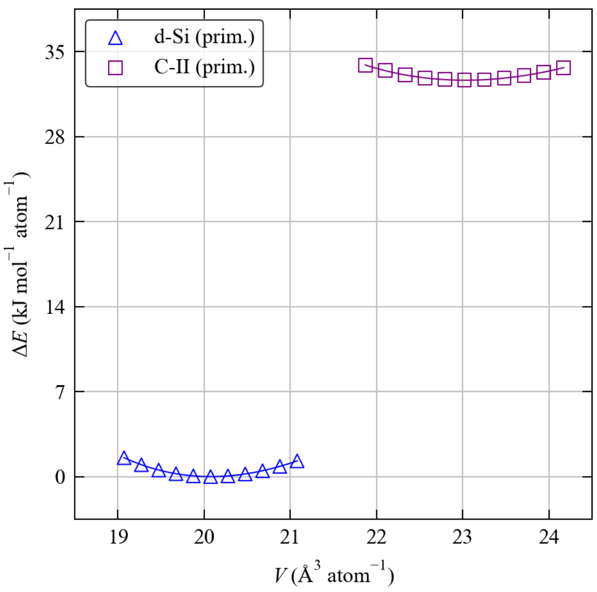

Figure S5. Energy/volume curves for the bulk diamond Si (d-SI) and Clathrate-II (C-II) structures calculated using the PBEsol functional with tightened convergence criteria (see text). Calculations were performed on the primitive cells of each structure. The markers show the calculated data points and the solid lines are fits to the Birch-Murnaghan equation of state (Equation 1).

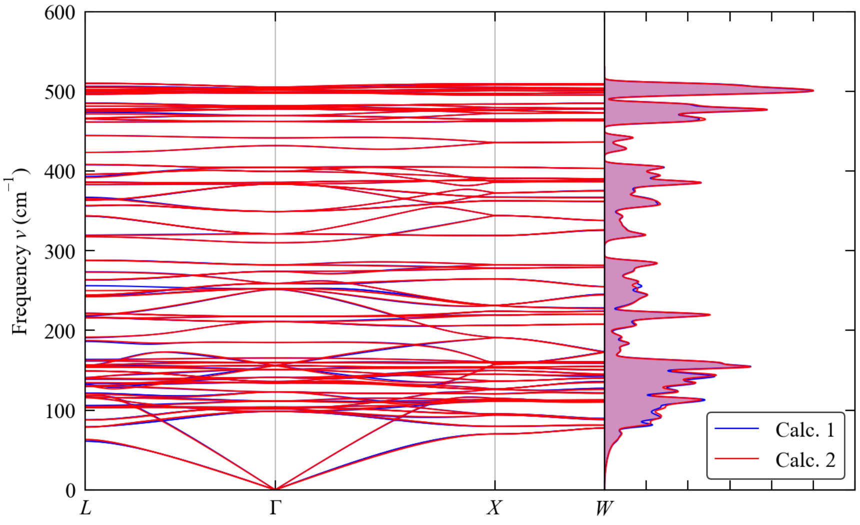

Figure S6. Comparison of the phonon dispersion and density of states curves for the Clathrate-II structure calculated from second-order force constants $\mathbf{\Phi}^{(2)}$ computed in a $2 \times 2 \times 2$ expansion of the primitive cell ("Calc. 1", blue) and in the (single) conventional unit cell ("Calc. 2", red).

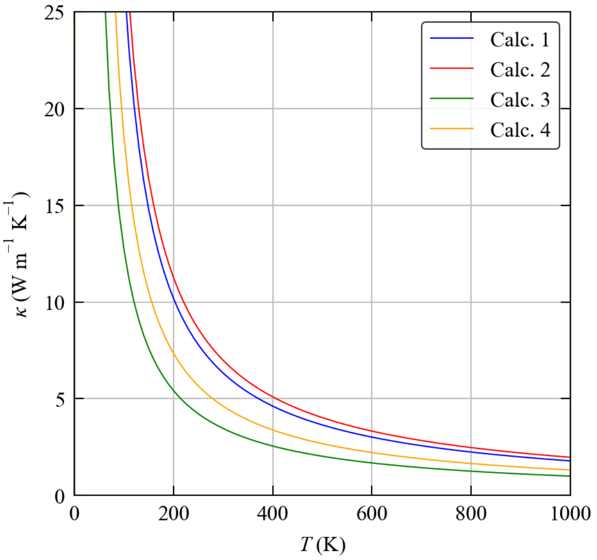

Figure S7. Comparison of the scalar average lattice thermal conductivity κ_{latt} as a function of temperature calculated for the Clathrate-II structure using: second-order force constants (FCs) $\mathbf{\Phi}^{(2)}$ computed in a $2 \times 2 \times 2$ expansion of the primitive cell and third-order FCs $\mathbf{\Phi}^{(3)}$ computed in the (single) conventional cell ("Calc. 1", blue); $\mathbf{\Phi}^{(2)}$ and $\mathbf{\Phi}^{(3)}$ computed in the conventional cell ("Calc. 2", red); and $\mathbf{\Phi}^{(2)}$ computed in a $2 \times 2 \times 2$ expansion of the primitive cell and $\mathbf{\Phi}^{(3)}$ computed in the conventional unit cell but with a range cutoff of 4.2 or 5 Å applied ("Calc. 3", orange and "Calc. 4", purple).

2. Peak Tables for Infrared (IR) and Raman spectra

Table S2. Calculated frequencies ν_j, irreducible representations (ir. reps), infrared activities I_j^{IR}, Raman activities $\bar{I}_j^{\mathrm{Raman}}$ and room-temperature (300 K) linewidths Γ_j for the $3n_a$ Γ-point vibrational modes of bulk diamond Si. The scalar Raman activities $\bar{I}^{\mathrm{Raman}}$ are calculated by performing a scalar average of the Raman tensors as defined in Equation 6 in the text. This data is used to construct the simulated spectra shown in Figure 4 in the text using Equation 2.

ν_j (cm^{-1})	Ir. Rep.	I_j^{IR} (e^2 amu$^{-\frac{1}{2}}$)	$\bar{I}_j^{\mathrm{Raman}}$ (10^3 Å^4 amu^{-1})	Γ_j (cm^{-1})
0.00	T1u	0.000	-	0.00
508.34	T2g	0.000	2.649	3.33

Table S3. Calculated frequencies ν_j, irreducible representations (ir. reps), infrared activities I_j^{IR}, Raman activities $\bar{I}_j^{\mathrm{Raman}}$ and room-temperature (300 K) linewidths Γ_j for the $3n_a$ Γ-point vibrational modes of the Clathrate-II (C-II) framework structure. The scalar Raman activities $\bar{I}^{\mathrm{Raman}}$ are calculated by performing a scalar average of the Raman tensors as defined in Equation 6 in the text. This data is used to construct the simulated spectra shown in Figure 4 in the text using Equation 2.

ν_j (cm^{-1})	Ir. Rep.	I_j^{IR} (e^2 amu$^{-\frac{1}{2}}$)	$\bar{I}_j^{\mathrm{Raman}}$ (10^3 Å^4 amu^{-1})	Γ_j (cm^{-1})
0.00	T1u	-	-	-
98.26	T2g	0.000	0.773	0.50
101.65	Eg	0.000	0.032	0.21
103.78	T1g	0.000	0.002	0.59
111.39	T2u	0.000	0.003	0.62
122.95	T1g	0.000	0.002	0.30
134.02	T1u	0.177	0.000	0.68
135.92	Eu	0.000	0.000	0.45
140.93	T2u	0.000	0.001	0.53
150.97	T2g	0.000	17.120	0.85
155.81	T1u	0.004	0.001	0.95
156.04	A1g	0.000	8.474	4.01
159.81	T1g	0.000	0.001	0.34
165.37	A2g	0.000	0.000	0.40
184.87	A2u	0.000	0.000	1.70
210.99	T2g	0.000	47.209	2.82
217.62	T1u	0.121	0.000	3.93
251.31	A2u	0.000	0.000	3.03
252.31	T1u	1.126	0.048	3.29
259.02	T2g	0.000	19.655	3.73
274.10	Eu	0.000	0.000	4.71
282.26	Eg	0.000	2.250	2.61
309.88	A1g	0.000	0.846	3.25
321.06	T2g	0.000	180.228	4.77
349.18	T1u	0.120	0.001	8.84
383.58	T2u	0.000	0.006	5.18
385.60	T2g	0.000	156.026	5.13
399.38	Eu	0.000	0.000	3.93
404.73	T1u	0.018	0.000	3.45
431.80	A2u	0.000	0.000	4.18
441.54	A1g	0.000	95.767	10.00
462.06	T1g	0.000	0.003	3.03
469.32	T2g	0.000	11.075	2.56
477.97	T1g	0.000	0.022	2.68
479.98	T2u	0.000	0.000	3.51
481.82	Eg	0.000	11.323	3.93
497.50	A1u	0.000	0.000	1.80
498.78	T1u	3.075	0.027	4.10
501.48	T2g	0.000	2.106	5.56
503.25	Eu	0.000	0.000	5.75
503.72	T2u	0.000	0.002	2.09
505.21	Eg	0.000	55.986	3.97

Table S4. Calculated frequencies ν_j, irreducible representations (ir. reps), infrared activities I_j^{IR}, Raman activities $\overline{I}_j^{\mathrm{Raman}}$ and room-temperature (300 K) linewidths Γ_j for the $3n_a$ Γ-point vibrational modes of the *o*C24 framework structure. The scalar Raman activities $\overline{I}^{\mathrm{Raman}}$ are calculated by performing a scalar average of the Raman tensors as defined in Equation 6 in the text. This data is used to construct the simulated spectra shown in Figure 4 in the text using Equation 2.

ν_j (cm^{-1})	Ir. Rep.	I_j^{IR} (e^2 amu$^{-\frac{1}{2}}$)	$\overline{I}_j^{\mathrm{Raman}}$ (10^3 Å^4 amu^{-1})	Γ_j (cm^{-1})
0.00	B2u	-	-	-
0.00	B1u	-	-	-
0.00	B3u	-	-	-
54.73	B1u	0.000	0.000	0.08
62.88	Au	0.000	0.000	0.12
80.03	Au	0.000	0.000	0.68
83.26	B3u	0.002	0.000	0.50
109.78	B2g	0.000	0.004	0.44
116.62	B1g	0.000	0.015	0.30
151.83	Ag	0.000	0.081	0.23
155.94	B2u	0.001	0.000	0.52
159.48	B3g	0.000	0.178	0.30
164.25	B2u	0.000	0.000	0.33
183.06	B1u	0.007	0.000	0.56
244.23	B3g	0.000	0.010	1.40
290.37	B1u	0.168	0.000	2.74
365.10	Ag	0.000	1.394	1.86
386.98	B3g	0.000	0.067	1.19
397.53	Ag	0.000	1.702	0.61
407.82	B2u	0.029	0.000	1.20
415.81	B1u	0.000	0.000	1.57
421.80	B3g	0.000	0.042	1.12
426.94	B2u	0.005	0.000	1.56
440.20	Ag	0.000	2.783	2.22
443.30	B2g	0.000	1.001	1.95
444.08	B1g	0.000	0.019	2.51
454.09	B3g	0.000	0.470	2.97
469.23	B3u	0.004	0.000	2.04
470.48	Ag	0.000	5.650	3.36
475.32	Au	0.000	0.000	2.43
479.41	B1g	0.000	1.092	2.40
491.32	B2g	0.000	0.925	2.26
496.49	B1u	0.008	0.000	1.25
504.58	B3g	0.000	0.001	2.73
524.36	Ag	0.000	4.379	3.58
528.69	B2u	0.002	0.000	1.67

Table S5. Calculated frequencies ν_j, irreducible representations (ir. reps), infrared activities I_j^{IR}, Raman activities $\bar{I}_j^{\mathrm{Raman}}$ and room-temperature (300 K) linewidths Γ_j for the $3n_a$ Γ-point vibrational modes of the K-II/C-I framework structure. The scalar Raman activities $\bar{I}^{\mathrm{Raman}}$ are calculated by performing a scalar average of the Raman tensors as defined in Equation 6 in the text. This data is used to construct the simulated spectra shown in Figure 4 in the text using Equation 2.

ν_j (cm^{-1})	Ir. Rep.	I_j^{IR} (e^2 amu$^{-\frac{1}{2}}$)	$\bar{I}_j^{\mathrm{Raman}}$ (10^3 Å^4 amu^{-1})	Γ_j (cm^{-1})
0.00	T1u	-	-	-
109.31	T2g	0.000	0.208	0.12
115.82	T1g	0.000	0.000	0.16
123.77	A2u	0.000	0.000	0.23
133.61	T1g	0.000	0.000	0.25
134.79	T2u	0.000	0.000	0.17
136.91	T1u	0.001	0.000	0.15
141.79	Eg	0.000	0.033	0.34
147.02	Eu	0.000	0.000	0.30
158.21	T1g	0.000	0.000	0.33
161.81	T2u	0.000	0.000	0.31
161.95	T2g	0.000	0.033	0.31
165.19	T1u	0.003	0.000	0.33
166.45	A2g	0.000	0.000	0.43
169.04	T2g	0.000	0.679	0.38
169.14	T1g	0.000	0.000	0.30
169.76	Eu	0.000	0.000	0.34
184.32	T1u	0.005	0.000	0.38
218.95	Eg	0.000	0.087	0.33
235.29	A2g	0.000	0.000	0.93
243.61	T2u	0.000	0.000	0.51
297.37	T1u	0.035	0.000	4.43
300.90	Eg	0.000	0.112	4.85
301.73	T2g	0.000	0.136	4.80
320.68	T2u	0.000	0.000	2.19
321.80	A1u	0.000	0.000	3.17
330.55	A1g	0.000	1.495	2.45
352.47	T1g	0.000	0.000	1.53
364.93	T2u	0.000	0.000	0.65
372.40	T1u	0.040	0.000	0.56
379.73	A2u	0.000	0.000	0.60
380.69	A2g	0.000	0.000	0.58
405.28	T2g	0.000	0.028	0.57
411.17	Eg	0.000	0.030	0.70
411.48	T1u	0.007	0.000	0.75
414.09	T1g	0.000	0.000	0.50
428.63	A1g	0.000	0.525	0.67
438.81	T2u	0.000	0.000	0.71
447.79	T1u	0.017	0.000	1.31
454.80	Eg	0.000	2.366	0.65
457.73	T2u	0.000	0.000	1.50
458.28	Eu	0.000	0.000	1.84
460.46	T1u	0.002	0.000	1.72
462.03	T2g	0.000	2.397	1.52
462.58	Eu	0.000	0.000	1.56
464.34	T1g	0.000	0.000	2.22
464.39	T2u	0.000	0.000	0.97
464.56	T2g	0.000	0.347	1.48
465.16	T1u	0.000	0.000	1.05
465.88	A1u	0.000	0.000	1.78

ν_j (cm^{-1})	Ir. Rep.	I_j^{IR} (e^2 amu$^{-\frac{1}{2}}$)	$\bar{I}_j^{\mathrm{Raman}}$ (10^3 Å^4 amu^{-1})	Γ_j (cm^{-1})
466.38	Eg	0.000	0.650	1.46
466.84	T1g	0.000	0.001	2.23
469.13	T2g	0.000	1.005	1.98
474.35	T2u	0.000	0.000	2.19
476.21	A1g	0.000	0.000	2.51
476.96	A2g	0.000	0.000	2.23
477.45	Eg	0.000	0.002	2.27

Table S6. Calculated frequencies ν_j, irreducible representations (ir. reps), infrared activities I_j^{IR}, Raman activities $\bar{I}_j^{\text{Raman}}$ and room-temperature (300 K) linewidths Γ_j for the $3n_a$ Γ-point vibrational modes of the K-V/C-VI framework structure. The scalar Raman activities $\bar{I}^{\text{Raman}}$ are calculated by performing a scalar average of the Raman tensors as defined in Equation 6 in the text. This data is used to construct the simulated spectra shown in Figure 4 in the text using Equation 2.

ν_j (cm^{-1})	Ir. Rep.	I_j^{IR} (e^2 amu$^{-\frac{1}{2}}$)	$\bar{I}_j^{\text{Raman}}$ (10^3 Å^4 amu^{-1})	Γ_j (cm^{-1})
0.00	B3u	-	-	0.00
0.00	B1u	-	-	0.00
0.00	B2u	-	-	0.00
111.51	B3g	0.000	0.144	0.11
111.51	B2g	0.000	0.144	0.11
116.72	B1g	0.000	0.092	0.20
116.78	Ag	0.000	0.088	0.20
119.98	B1g	0.000	0.000	0.18
121.70	B2g	0.000	0.017	0.15
121.76	B3g	0.000	0.017	0.15
124.59	B3g	0.000	0.000	0.13
135.88	B3u	0.003	0.000	0.14
135.89	B1u	0.000	0.000	0.22
135.90	Au	0.000	0.000	0.22
135.91	B2u	0.003	0.000	0.14
139.10	Au	0.000	0.000	0.30
145.78	B3u	0.000	0.000	0.09
146.11	Ag	0.000	0.024	0.29
146.11	B1g	0.000	0.023	0.29
147.87	B2g	0.000	0.000	0.27
155.97	B3u	0.004	0.000	0.33
155.99	B2u	0.004	0.000	0.39
156.50	B2g	0.000	0.002	0.30
156.52	B3g	0.000	0.002	0.30
158.49	B1u	0.002	0.000	0.56
158.71	Au	0.000	0.000	0.46
158.74	B1u	0.000	0.000	0.47
160.50	B1g	0.000	0.000	0.41
163.65	B3g	0.000	0.000	0.67
164.62	B2g	0.000	0.130	0.35
164.65	B3g	0.000	0.129	0.36
167.20	B1g	0.000	0.068	0.42
167.21	Ag	0.000	0.074	0.43
169.91	Au	0.000	0.000	0.49
169.91	B1u	0.000	0.000	0.50
169.93	B1g	0.000	0.000	0.47
170.20	B3u	0.000	0.000	0.60
170.22	B2u	0.000	0.000	0.59
174.56	B2g	0.000	0.000	0.24
175.93	B2u	0.000	0.000	0.65
194.15	B3u	0.000	0.000	0.37
212.52	Ag	0.000	2.086	0.43
232.15	B1u	0.011	0.000	0.39
244.29	B1g	0.000	0.000	0.89
244.33	Ag	0.000	0.001	0.87
257.75	B2u	0.001	0.000	0.90
257.77	B3u	0.001	0.000	0.89
272.54	B2u	0.000	0.000	0.99
286.39	Ag	0.000	0.004	3.08
294.66	Au	0.000	0.000	3.24

ν_j (cm^{-1})	Ir. Rep.	I_j^{IR} (e^2 amu$^{-\frac{1}{2}}$)	$\overline{I}_j^{\mathrm{Raman}}$ (10^3 Å^4 amu^{-1})	Γ_j (cm^{-1})
294.67	B1u	0.000	0.000	3.25
307.93	B2u	0.001	0.000	2.62
307.97	B3u	0.001	0.000	2.61
321.09	B2g	0.000	0.038	3.56
321.10	B3g	0.000	0.041	3.59
324.70	Ag	0.000	1.172	6.71
329.18	B3g	0.000	0.000	4.72
332.22	Ag	0.000	0.024	4.44
332.24	B1g	0.000	0.021	4.28
350.99	B1u	0.017	0.000	1.17
361.91	B1u	0.000	0.000	1.42
361.91	Au	0.000	0.000	1.46
377.56	B2u	0.017	0.000	0.71
377.63	B3u	0.017	0.000	0.70
379.86	Ag	0.000	0.011	0.93
379.91	B1g	0.000	0.010	0.92
382.20	B2u	0.000	0.000	0.98
383.12	B3u	0.000	0.000	0.63
388.98	Ag	0.000	0.132	0.85
394.64	B2g	0.000	0.000	0.55
399.68	B2u	0.005	0.000	0.87
399.72	B3u	0.005	0.000	0.87
403.46	B2u	0.000	0.000	0.95
410.52	B3g	0.000	0.000	0.65
411.81	B1g	0.000	0.044	0.73
411.85	Ag	0.000	0.041	0.69
411.95	B2g	0.000	0.177	0.68
411.98	B3g	0.000	0.179	0.66
413.55	B1u	0.000	0.000	0.40
429.88	B2u	0.000	0.000	0.42
440.15	B3u	0.000	0.000	0.68
440.41	Ag	0.000	0.145	1.50
440.42	B1g	0.000	0.149	1.53
440.61	Au	0.000	0.000	0.77
440.63	B1u	0.000	0.000	0.76
445.28	Ag	0.000	0.316	1.08
445.28	B1u	0.000	0.000	0.87
450.93	B2u	0.000	0.000	1.76
450.95	B3u	0.000	0.000	1.71
452.09	B1u	0.000	0.000	1.51
452.15	Au	0.000	0.000	1.50
459.00	B1g	0.000	0.051	1.46
459.04	Ag	0.000	0.049	1.46
459.05	Au	0.000	0.000	1.63
459.10	B1u	0.000	0.000	1.65
460.44	Au	0.000	0.000	3.16
461.21	B2g	0.000	0.673	2.08
461.25	Ag	0.000	1.109	1.46
461.32	B3g	0.000	0.661	2.09
461.43	B2g	0.000	0.005	2.00
463.07	B1u	0.000	0.000	2.00
464.10	B2g	0.000	0.323	1.40
464.11	B3g	0.000	0.342	1.40
464.44	B1g	0.000	0.000	2.96
464.88	B3u	0.007	0.000	2.37
464.91	B2u	0.007	0.000	2.39
467.38	B3g	0.000	0.000	2.01

ν_j (cm^{-1})	Ir. Rep.	I_j^{IR} (e^2 amu$^{-\frac{1}{2}}$)	$\bar{I}_j^{\mathrm{Raman}}$ (10^3 Å^4 amu^{-1})	Γ_j (cm^{-1})
485.14	Au	0.000	0.000	2.39
485.24	B1u	0.000	0.000	2.39
485.55	B3u	0.000	0.000	2.36
485.66	B2u	0.000	0.000	2.31
486.02	Ag	0.000	1.716	1.79
486.06	B1g	0.000	1.683	1.77
486.76	B2g	0.000	0.012	2.54
486.90	B3g	0.000	0.010	2.57
492.33	B2u	0.000	0.000	4.88
492.87	Ag	0.000	0.003	4.55
492.92	B1g	0.000	0.005	4.65
498.20	Ag	0.000	1.424	5.05
500.53	B3u	0.000	0.000	3.04

Table S7. Calculated frequencies ν_j, irreducible representations (ir. reps), infrared activities I_j^{IR}, Raman activities $\overline{I}_j^{\mathrm{Raman}}$ and room-temperature (300 K) linewidths Γ_j for the $3n_a$ Γ-point vibrational modes of the K-VII/C-V framework structure. The scalar Raman activities $\overline{I}^{\mathrm{Raman}}$ are calculated by performing a scalar average of the Raman tensors as defined in Equation 6 in the text. This data is used to construct the simulated spectra shown in Figure 4 in the text using Equation 2.

ν_j (cm^{-1})	Ir. Rep.	I_j^{IR} (e^2 amu$^{-\frac{1}{2}}$)	$\overline{I}_j^{\mathrm{Raman}}$ (10^3 Å^4 amu^{-1})	Γ_j (cm^{-1})
-0.01	A2u	-	-	-
0.00	E1u	0.000	-	-
78.70	E2g	0.000	0.000	0.07
91.66	E2u	0.000	0.000	0.06
113.43	B2g	0.000	0.000	0.18
118.40	E2g	0.000	0.335	0.17
120.15	E1g	0.000	0.386	0.20
121.04	E1u	0.000	0.001	0.21
125.94	A2g	0.000	0.000	0.13
126.44	E1g	0.000	0.065	0.14
127.86	B1g	0.000	0.000	0.14
129.06	E2u	0.000	0.000	0.15
132.27	B2u	0.000	0.000	0.13
132.88	A2u	0.001	0.000	0.17
137.95	E2g	0.000	0.019	0.21
139.31	E1g	0.000	0.029	0.19
143.01	E1u	0.000	0.000	0.14
143.46	A1u	0.000	0.000	0.13
145.87	A2g	0.000	0.000	0.23
147.11	B2g	0.000	0.000	0.12
153.61	E2u	0.000	0.000	0.28
154.25	E2g	0.000	0.001	0.26
154.96	B1g	0.000	0.000	0.27
157.67	E1u	0.000	0.000	0.31
160.34	B2u	0.000	0.000	0.28
160.37	B2g	0.000	0.000	0.48
162.38	A1u	0.000	0.000	0.35
162.41	A2u	0.000	0.000	0.46
162.77	E1g	0.000	0.063	0.40
162.93	E2u	0.000	0.000	0.40
163.21	B1u	0.000	0.000	0.15
164.02	E1u	0.001	0.000	0.37
164.55	E2g	0.000	0.014	0.37
167.81	E2u	0.000	0.000	0.37
168.04	A2g	0.000	0.000	0.28
168.48	E1g	0.000	0.005	0.32
168.82	E2g	0.000	0.008	0.35
169.56	E1g	0.000	0.051	0.38
170.45	B2u	0.000	0.000	0.27
170.52	B1g	0.000	0.000	0.26
170.62	B2g	0.000	0.000	0.34
173.57	A2g	0.000	0.000	0.23
173.72	A1g	0.000	0.140	0.49
173.99	A2u	0.002	0.000	0.35
182.15	E1u	0.002	0.001	0.38
182.48	E2u	0.000	0.000	0.37
183.58	B1u	0.000	0.000	0.42
244.01	E2g	0.000	0.113	0.84
259.94	A1g	0.000	0.732	0.53
262.49	E1u	0.022	0.001	0.86

ν_j (cm^{-1})	Ir. Rep.	I_j^{IR} (e^2 amu$^{-\frac{1}{2}}$)	$\bar{I}_j^{Raman}$ (10^3 Å^4 amu^{-1})	Γ_j (cm^{-1})
266.80	A2u	0.007	0.000	0.67
267.44	E1g	0.000	0.090	0.60
268.45	A1g	0.000	0.230	0.48
274.17	B1u	0.000	0.001	1.10
276.12	E2u	0.000	0.000	1.53
287.60	E1u	0.000	0.003	3.52
294.75	B1u	0.000	0.001	3.30
301.85	E2g	0.000	0.070	3.63
315.30	A1g	0.000	1.100	4.15
317.35	B2g	0.000	0.000	3.79
327.87	A2u	0.002	0.000	5.02
330.33	E1g	0.000	0.034	2.30
331.32	E2u	0.000	0.000	2.39
341.02	E2g	0.000	0.002	1.97
347.45	E1u	0.007	0.001	1.51
354.41	B2g	0.000	0.000	1.17
356.22	B1u	0.000	0.000	1.07
357.35	A1u	0.000	0.000	0.87
360.93	B2u	0.000	0.000	0.74
365.21	E2g	0.000	0.067	0.59
368.30	E1u	0.004	0.000	0.63
370.51	E2u	0.000	0.000	0.78
375.38	A1g	0.000	0.023	0.68
376.21	E1g	0.000	0.030	0.48
380.39	A2u	0.005	0.000	0.53
383.61	B1u	0.000	0.000	0.56
395.60	B2g	0.000	0.000	0.70
396.45	A1g	0.000	0.012	0.60
398.92	A2u	0.000	0.000	0.71
404.24	E1u	0.006	0.001	0.65
405.19	E2g	0.000	0.053	0.56
406.04	E1g	0.000	0.006	0.49
406.21	B1u	0.000	0.000	0.45
408.09	B2g	0.000	0.000	0.43
413.56	A2u	0.000	0.000	0.26
419.47	E2g	0.000	0.025	0.44
419.75	E2u	0.000	0.000	0.67
420.66	B1g	0.000	0.000	0.48
421.58	A2g	0.000	0.000	0.46
422.89	B1u	0.000	0.001	0.64
424.43	E1u	0.001	0.001	0.67
424.59	A1g	0.000	0.889	0.42
442.65	E2u	0.000	0.000	1.17
444.86	B2g	0.000	0.000	0.98
447.09	E1u	0.000	0.001	1.18
448.17	A2u	0.003	0.000	1.41
449.95	E1g	0.000	0.456	1.45
452.05	E2g	0.000	0.316	1.30
453.33	E1u	0.000	0.000	1.41
454.49	A1u	0.000	0.000	1.40
455.68	E2u	0.000	0.000	1.49
455.69	A1g	0.000	0.716	1.02
456.42	B2u	0.000	0.000	1.28
456.57	A1g	0.000	2.293	1.37
456.73	B1u	0.000	0.000	1.38
457.06	E2u	0.000	0.000	1.59
458.24	E1g	0.000	1.280	1.51

ν_j (cm^{-1})	Ir. Rep.	I_j^{IR} (e^2 amu$^{-\frac{1}{2}}$)	$\bar{I}_j^{Raman}$ (10^3 Å^4 amu^{-1})	Γ_j (cm^{-1})
459.09	E2u	0.000	0.000	1.89
461.03	B1u	0.000	0.000	1.43
461.19	E1u	0.000	0.000	1.85
461.48	E1g	0.000	0.438	1.54
463.42	E2g	0.000	0.994	1.75
463.54	B2g	0.000	0.000	1.90
465.16	A2u	0.005	0.000	1.92
467.57	E2g	0.000	1.824	1.35
467.73	B1g	0.000	0.000	2.32
468.73	E1g	0.000	1.450	1.64
470.67	A2g	0.000	0.000	2.44
471.38	A1g	0.000	1.340	1.80
471.93	B2g	0.000	0.000	2.17
472.06	E1u	0.000	0.000	1.49
472.96	E2g	0.000	0.178	2.07
473.92	B2u	0.000	0.000	1.53
474.02	A1u	0.000	0.000	1.65
474.60	E1g	0.000	0.258	1.86
475.07	E2u	0.000	0.000	1.43
476.65	E2g	0.000	0.572	1.69
477.80	E1u	0.000	0.000	1.12
477.97	E2u	0.000	0.000	1.55
480.02	E1u	0.001	0.001	2.15
480.12	E1g	0.000	0.087	2.12
480.70	A2g	0.000	0.000	1.38
481.58	E2g	0.000	0.071	2.25
481.91	B2u	0.000	0.000	1.32
485.27	B1u	0.000	0.000	3.40
485.68	A1g	0.000	0.088	2.65

3. Analysis of the Phonon Linewidths

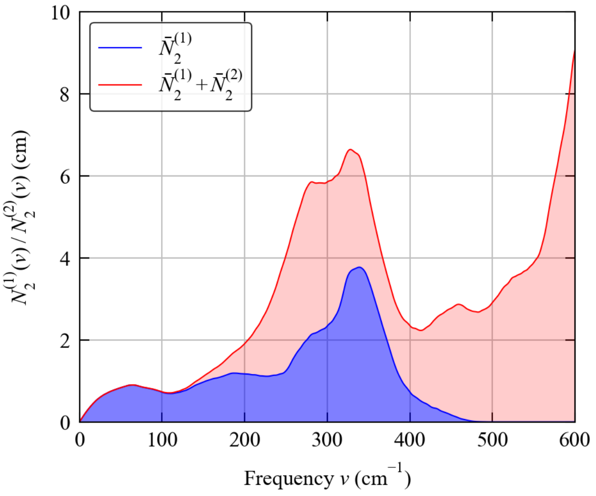

Figure S8. Unscaled weighted two-phonon joint density of states (w-JDoS) functions $\bar{N}_2(\nu) = \bar{N}_2^{(1)}(\nu) + \bar{N}_2^{(2)}(\nu)$ (Equation 22 in the text) for bulk diamond Si (d-Si) at T = 300 K.

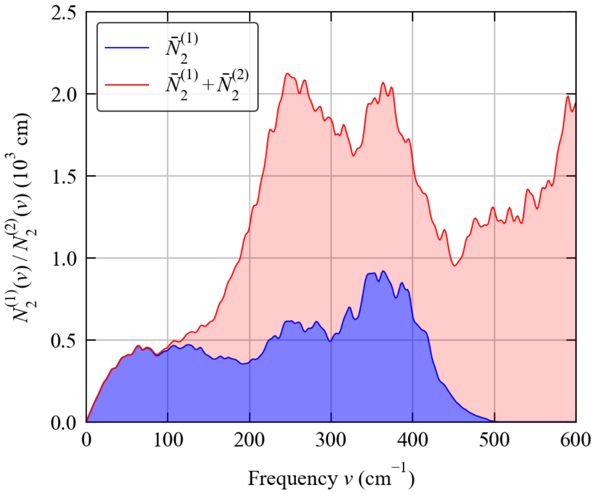

Figure S9. Unscaled weighted two-phonon joint density of states (w-JDoS) functions $\bar{N}_2(\nu) = \bar{N}_2^{(1)}(\nu) + \bar{N}_2^{(2)}(\nu)$ (Equation 22 in the text) for the Clathrate-II (C-II) structure at T = 300 K.

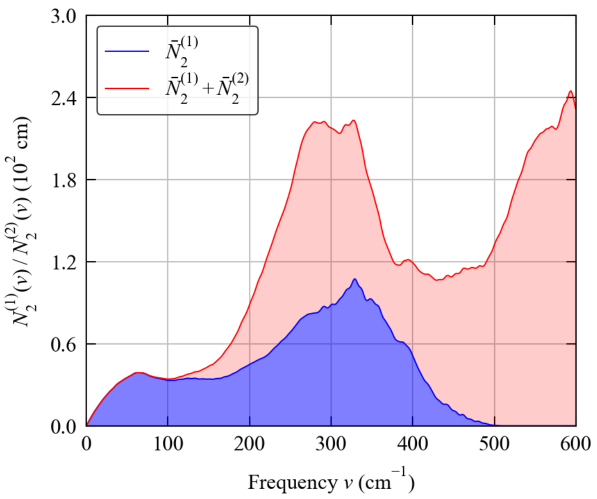

Figure S10. Unscaled weighted two-phonon joint density of states (w-JDoS) functions $\bar{N}_2(\nu) = \bar{N}_2^{(1)}(\nu) + \bar{N}_2^{(2)}(\nu)$ (Equation 22 in the text) for the *o*C24 structure at T = 300 K.

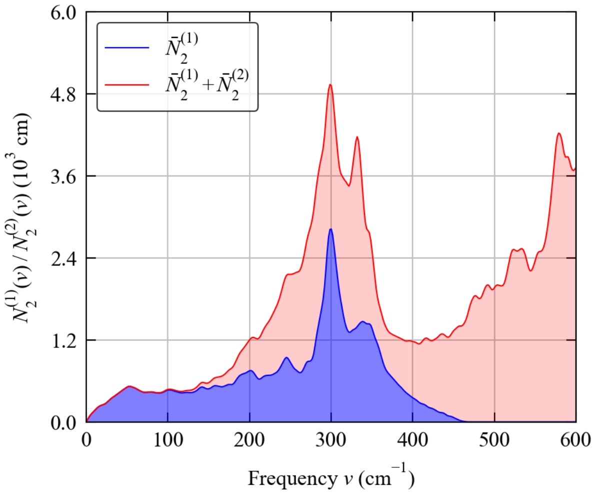

Figure S11. Unscaled weighted two-phonon joint density of states (w-JDoS) functions $\bar{N}_2(\nu) = \bar{N}_2^{(1)}(\nu) + \bar{N}_2^{(2)}(\nu)$ (Equation 22 in the text) for the K-II/C-I structure at T = 300 K.

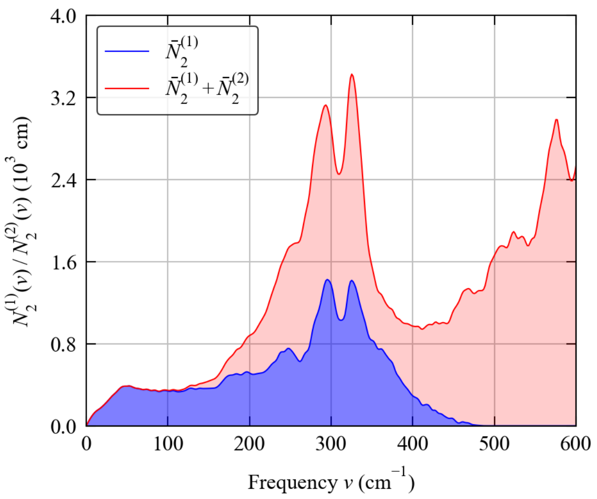

Figure S12. Unscaled weighted two-phonon joint density of states (w-JDoS) functions $\bar{N}_2(\nu) = \bar{N}_2^{(1)}(\nu) + \bar{N}_2^{(2)}(\nu)$ (Equation 22 in the text) for the K-V/C-VI structure at T = 300 K.

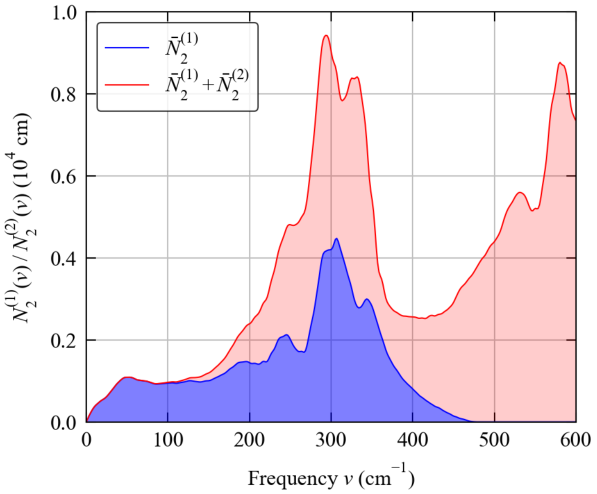

Figure S13. Unscaled weighted two-phonon joint density of states (w-JDoS) functions $\bar{N}_2(\nu) = \bar{N}_2^{(1)}(\nu) + \bar{N}_2^{(2)}(\nu)$ (Equation 22 in the text) for the K-VII/C-V structure at T = 300 K.

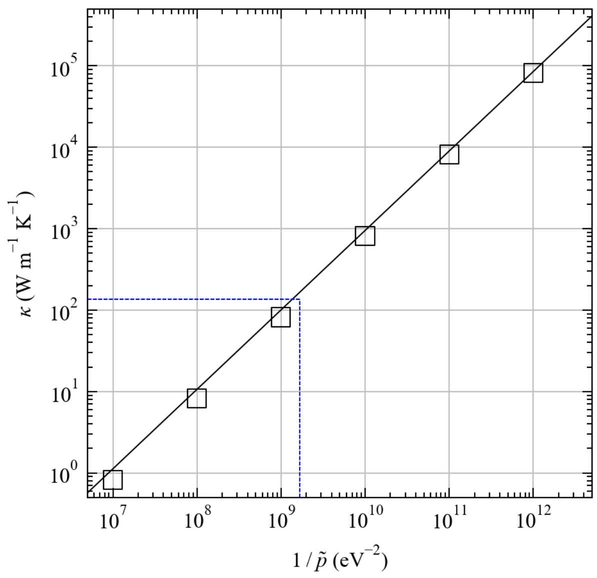

Figure S14. Lattice thermal conductivity κ_{latt} of bulk diamond Si (d-Si) at T = 300 K obtained by replacing the squared three-phonon interaction strengths $|\Phi_{-qj,q'j',q''j''}|^2$ in Equation 17 in the text with a constant value $\tilde{P}$ as in the model in Equation 18. The markers show the data points, the black line is a linear fit to the data, and the dashed blue lines mark the value of $\tilde{P}$ required to obtain the 300 K κ_{latt} calculated from the "full" single-mode relaxation-time approximation model.

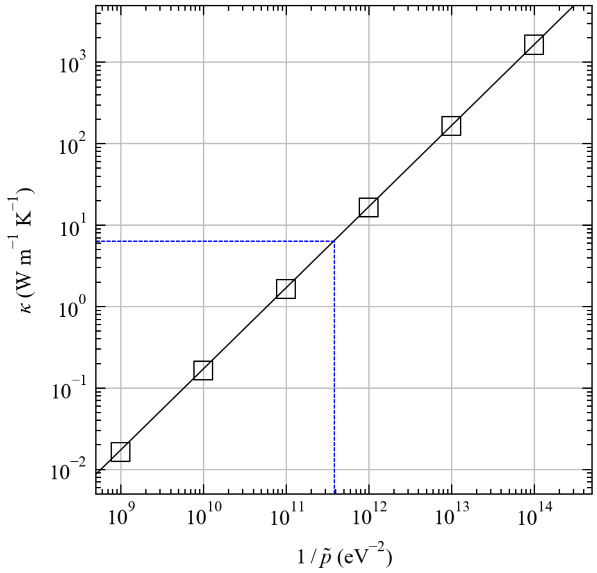

Figure S15. Lattice thermal conductivity κ_{latt} of the Clathrate-II (C-II) framework structure at T = 300 K obtained by replacing the squared three-phonon interaction strengths $|\Phi_{-qj,q'j',q''j''}|^2$ in Equation 17 in the text with a constant value $\tilde{P}$ as in the model in Equation 18. The markers show the data points, the black line is a linear fit to the data, and the dashed blue lines mark the value of $\tilde{P}$ required to obtain the 300 K κ_{latt} calculated from the "full" single-mode relaxation-time approximation model.

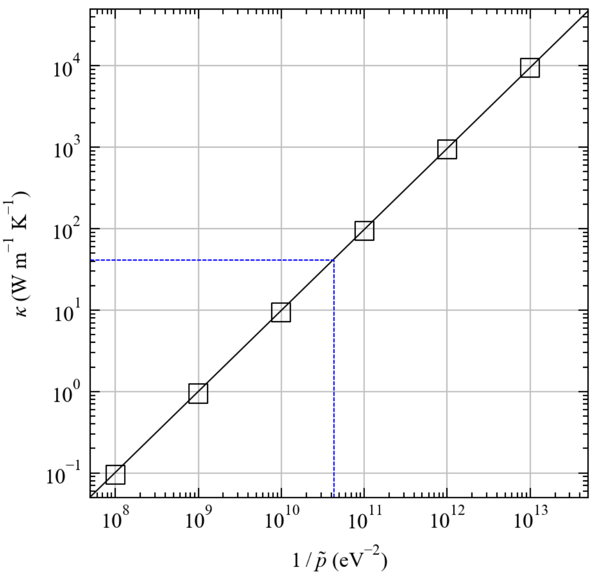

Figure S16. Lattice thermal conductivity κ_{latt} of the *o*C24 framework structure at T = 300 K obtained by replacing the squared three-phonon interaction strengths $|\Phi_{-qj,q'j',q''j''}|^2$ in Equation 17 in the text with a constant value $\tilde{P}$ as in the model in Equation 18. The markers show the data points, the black line is a linear fit to the data, and the dashed blue lines mark the value of $\tilde{P}$ required to obtain the 300 K κ_{latt} calculated from the "full" single-mode relaxation-time approximation model.

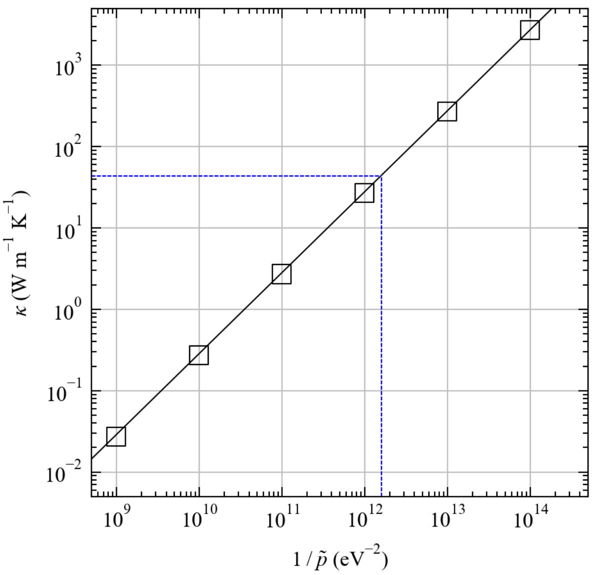

Figure S17. Lattice thermal conductivity κ_{latt} of the K-II/C-I framework structure at T = 300 K obtained by replacing the squared three-phonon interaction strengths $|\Phi_{-qj,q'j',q''j''}|^2$ in Equation 17 in the text with a constant value $\tilde{P}$ as in the model in Equation 18. The markers show the data points, the black line is a linear fit to the data, and the dashed blue lines mark the value of $\tilde{P}$ required to obtain the 300 K κ_{latt} calculated from the "full" single-mode relaxation-time approximation model.

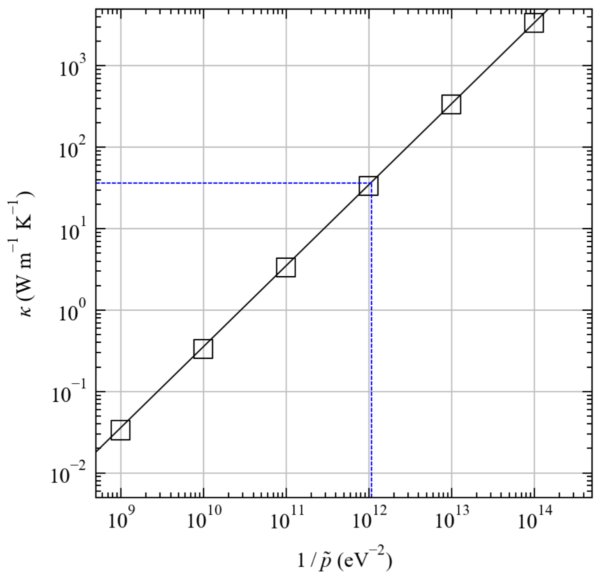

Figure S18. Lattice thermal conductivity κ_{latt} of the K-V/C-VI framework structure at T = 300 K obtained by replacing the squared three-phonon interaction strengths $|\Phi_{-qj,q'j',q''j''}|^2$ in Equation 17 in the text with a constant value $\tilde{P}$ as in the model in Equation 18. The markers show the data points, the black line is a linear fit to the data, and the dashed blue lines mark the value of $\tilde{P}$ required to obtain the 300 K κ_{latt} calculated from the "full" single-mode relaxation-time approximation model.

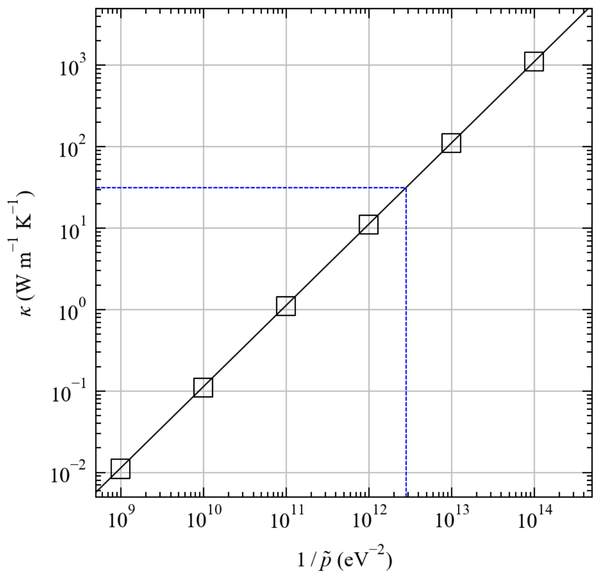

Figure S19. Lattice thermal conductivity κ_{latt} of the K-VII/C-V framework structure at T = 300 K obtained by replacing the squared three-phonon interaction strengths $|\Phi_{-qj,q'j',q''j''}|^2$ in Equation 17 in the text with a constant value $\tilde{P}$ as in the model in Equation 18. The markers show the data points, the black line is a linear fit to the data, and the dashed blue lines mark the value of $\tilde{P}$ required to obtain the 300 K κ_{latt} calculated from the "full" single-mode relaxation-time approximation model.

References

1. Dong, J.; Sankey, O.F.; Kern, G. Theoretical study of the vibrational modes and their pressure dependence in the pure clathrate-II silicon framework. *Phys. Rev. B* **1999**, *60*, 950–958. doi:10.1103/PhysRevB.60.950.
2. Zhao, H.Y.; Wang, J.; Ma, Q.M.; Liu, Y. sp_3-Bonded silicon allotropes based on the Kelvin problem. *Phys. Chem. Chem. Phys.* **2013**, *15*, 17619–17625. doi:10.1039/C3CP50946J.
3. Härkönen, V.J.; Karttunen, A.J. *Ab initio* studies on the lattice thermal conductivity of silicon clathrate frameworks II and VIII. *Phys. Rev. B* **2016**, *93*, 024307. doi:10.1103/PhysRevB.93.024307.
4. Norouzzadeh, P.; Krasinski, J.S.; Tadano, T. Thermal conductivity of type-I, type-II, and type-VIII pristine silicon clathrates: A first-principles study. *Phys. Rev. B* **2017**, *96*, 245201. doi:10.1103/PhysRevB.96.245201.
5. Ceperley, D.M.; Alder, B.J. Ground State of the Electron Gas by a Stochastic Method. *Phys. Rev. Lett.* **1980**, *45*, 566–569. doi:10.1103/PhysRevLett.45.566.
6. Perdew, J.P.; Chevary, J.A.; Vosko, S.H.; Jackson, K.A.; Pederson, M.R.; Singh, D.J.; Fiolhais, C. Atoms, molecules, solids, and surfaces: Applications of the generalized gradient approximation for exchange and correlation. *Phys. Rev. B* **1992**, *46*, 6671–6687. doi:10.1103/PhysRevB.46.6671.
7. Perdew, J.P.; Burke, K.; Ernzerhof, M. Generalized Gradient Approximation Made Simple. *Phys. Rev. Lett.* **1996**, *77*, 3865–3868. doi:10.1103/PhysRevLett.77.3865.
8. Perdew, J.P.; Ruzsinszky, A.; Csonka, G.I.; Vydrov, O.A.; Scuseria, G.E.; Constantin, L.A.; Zhou, X.; Burke, K. Restoring the Density-Gradient Expansion for Exchange in Solids and Surfaces. *Phys. Rev. Lett.* **2008**, *100*, 136406. doi:10.1103/PhysRevLett.100.136406.
9. Skelton, J.M.; Tiana, D.; Parker, S.C.; Togo, A.; Tanaka, I.; Walsh, A. Influence of the exchange-correlation functional on the quasi-harmonic lattice dynamics of II-VI semiconductors. *The Journal of Chemical Physics* **2015**, *143*, 064710. doi:10.1063/1.4928058.
10. Jain, A.; Ong, S.P.; Hautier, G.; Chen, W.; Richards, W.D.; Dacek, S.; Cholia, S.; Gunter, D.; Skinner, D.; Ceder, G.; et al. The Materials Project: A materials genome approach to accelerating materials innovation. *APL Materials* **2013**, *1*, 011002. doi:10.1063/1.4812323.
11. Blöchl, P.E. Projector augmented-wave method. *Phys. Rev. B* **1994**, *50*, 17953–17979. doi:10.1103/PhysRevB.50.17953.
12. Kresse, G.; Joubert, D. From ultrasoft pseudopotentials to the projector augmented-wave method. *Phys. Rev. B* **1999**, *59*, 1758–1775. doi:10.1103/PhysRevB.59.1758.
13. Murnaghan, F.D. The Compressibility of Media under Extreme Pressures. *Proceedings of the National Academy of Sciences* **1944**, *30*, 244–247. doi:10.1073/pnas.30.9.244.
14. Birch, F. Finite Elastic Strain of Cubic Crystals. *Phys. Rev.* **1947**, *71*, 809–824. doi:10.1103/PhysRev.71.809.
15. Nolas, G.S.; Beekman, M.; Gryko, J.; Lamberton, G.A.; Tritt, T.M.; McMillan, P.F. Thermal conductivity of elemental crystalline silicon clathrate Si_{136}. *Applied Physics Letters* **2003**, *82*, 910–912. doi:10.1063/1.1544640.
16. Beekman, M.; Nolas, G. Synthesis and thermal conductivity of type II silicon clathrates. *Physica B: Condensed Matter* **2006**, *383*, 111–114. doi:10.1016/j.physb.2006.03.070.
